# *Batzella*, *Crambe* and *Monanchora*: Highly Prolific Marine Sponge Genera Yielding Compounds with Potential Applications for Cancer and Other Therapeutic Areas

**DOI:** 10.3390/nu10010033

**Published:** 2018-01-02

**Authors:** Amr El-Demerdash, Atanas G. Atanasov, Anupam Bishayee, Mamdouh Abdel-Mogib, John N. A. Hooper, Ali Al-Mourabit

**Affiliations:** 1 Institut de Chimie des Substances Naturelles, CNRS UPR 2301, Univ. Paris-Sud, University of Paris-Saclay, 1, Avenue de la Terrasse, 91198 Gif-Sur-Yvette, France; Ali.ALMOURABIT@cnrs.fr; 2 Organic Chemistry Division, Chemistry Department, Faculty of Science, Mansoura University, Mansoura 35516, Egypt; mmdhbdlmgb@gmail.com; 3 Institute of Genetics and Animal Breeding of the Polish Academy of Sciences, 05-552 Jastrzebiec, Poland; 4 Department of Pharmacognosy, University of Vienna, 1090 Vienna, Austria; 5 Department of Pharmaceutical Sciences, College of Pharmacy, Larkin University, 18301 N. Miami Avenue, Miami, FL 33169, USA; 6 Queensland Museum, P.O. Box 3300, South Brisbane, QLD BC 4101, Australia; john.hooper@qm.qld.gov.au

**Keywords:** marine sponges, Poecilosclerida, Batzella, *Crambe*, *Monanchora*, guanidine alkaloids, pyrroloquinoline alkaloids, bioactivities, biomimetic synthesis

## Abstract

Pyrroloquinoline and guanidine-derived alkaloids present distinct groups of marine secondary metabolites with structural diversity that displayed potentialities in biological research. A considerable number of these molecular architectures had been recorded from marine sponges belonging to different marine genera, including *Batzella*, *Crambe*, *Monanchora*, *Clathria*, *Ptilocaulis* and New Caledonian starfishes *Fromia monilis* and *Celerina heffernani*. In this review, we aim to comprehensively cover the chemodiversity and the bioactivities landmarks centered around the chemical constituents exclusively isolated from these three marine genera including *Batzella*, *Crambe* and *Monanchora* over the period 1981–2017, paying a special attention to the polycyclic guanidinic compounds and their proposed biomimetic landmarks. It is concluded that these marine sponge genera represent a rich source of novel compounds with potential applications for cancer and other therapeutic areas.

## 1. Introduction

As a result of the rise of many current medical challenges, including hepatitis, parasitic infection, lifestyle-induced diseases, such as diabetes, hypertension, many forms of cancer, multi-drug resistance pathogens and other diseases, searching for new bioactive compounds with novel modes of action is necessary. Marine natural products represent potent, promising and sustainable sources for biomedications [1]. Up to present time, eight marine-derived drugs were approved for market pipelines for the treatment of some of these current medical challenges [2,3]. Marine sponges (phylum Porifera), even though they are the most primitive class within the animal kingdom, are considered renewable powerful suppliers for bioactives. The marine genera belonging to the order Poecilosclerida, *Batzella* (family Chondropsidae), *Crambe* and *Monanchora* (family Crambeidae), are rich in the production of highly physiologically active pyrroloquinoline and guanidine-derived alkaloids [4,5,6], with a vast scope of biological potentialities including cytotoxic and antiviral [7,8,9,10,11,12], HIV-1 inhibitors [13,14], enzyme inhibitors [15], receptor antagonist [16], Ca^2+^ channel blocker [17], antifungal [18] and antimicrobial [19,20,21]. These interesting compounds are considered taxonomic markers in particular for some Poecilosclerida and Axinellida marine sponge genera [5]. Their complex molecular architectures and potent biological activities have made them for years ideal target molecules for synthetic applications [22,23,24,25,26,27,28]. Beside the production of guanidine-derived architectures, some deep-water species of *Batzella* produced pyrroloquinoline-derived alkaloids, which raises a chemotaxonomic question about the systematic relatedness of this genus (family Chondropsidae) to other genera like *Crambe* and *Monanchora* (family Crambeidae). A chemosystematic exploration has revealed that *Batzella* sponges containing cyclic guanidine alkaloids are chemically and taxonomically similar, and perhaps synonymous with, *Monanchora* and *Crambe*. However, the deep-water *Batzella* sponges produced pyrroloquinoline alkaloids is taxonomically unrelated to the *Batzella* previously mentioned. Chemically, it is almost similar to the *Zyzzya* and *Latrunculia* marine sponges but their phylogenetic relationship is still undetermined [29]. Systematically, the World Porifera Database accepts nine valid species of *Batzella* [30], nine valid species in the genus *Crambe* [31] and fourteen valid species currently in the genus *Monanchora* [32]. To the best of our knowledge, previous chemical investigations of *Batzella* was centered on only a single unidentified species from Madagascar [33], for the genus *Crambe* only one identified species, the type species *Crambe crambe* from the Mediterranean [34] and finally five identified *Monanchora* species including *Monanchora ungiculata* [35], *Monanchora dianchora* [36], *Monanchora pulchra* [37], *Monanchora arbuscula* [38] and *Monanchora unguifera* [35] in addition to one unidentified species of *Monanchora* n. sp. [39]. 

## 2. Chemistry and Biology of Natural Products Isolated from *Batzella*, *Crambe* and *Monanchora*

In this review, we provide comprehensive insights on the previous chemical and biological reports for the metabolites of the three marine genera. To facilitate the handling of this survey, the isolated natural compounds are classified by their polycyclic skeleton coupled with their recorded biological potentialities whenever applicable.

### 2.1. Piperidine Iminosugars Alkaloids

**Figure 1:**
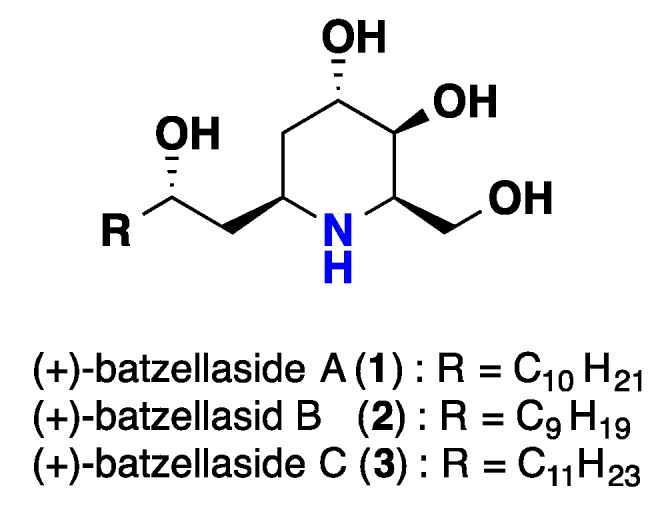
Isolated iminosugars **1**–**3** from *Batzella* sp.

(+)-Batzellasides A-C (**1**–**3**), three alkylated piperidine iminosugars were isolated from a Madagascar sponge, *Batzella* sp. and represented the first naturally occurring marine iminosugars. These compounds demonstrated inhibition of the growth of *Staphylococcus epidermidis* with MICs (Minimum Inhibitory Concentration) that were under 6.3 μM [33] (Figure 1).

### 2.2. Bicyclic Guanidine Alkaloids

**Figure 2:**
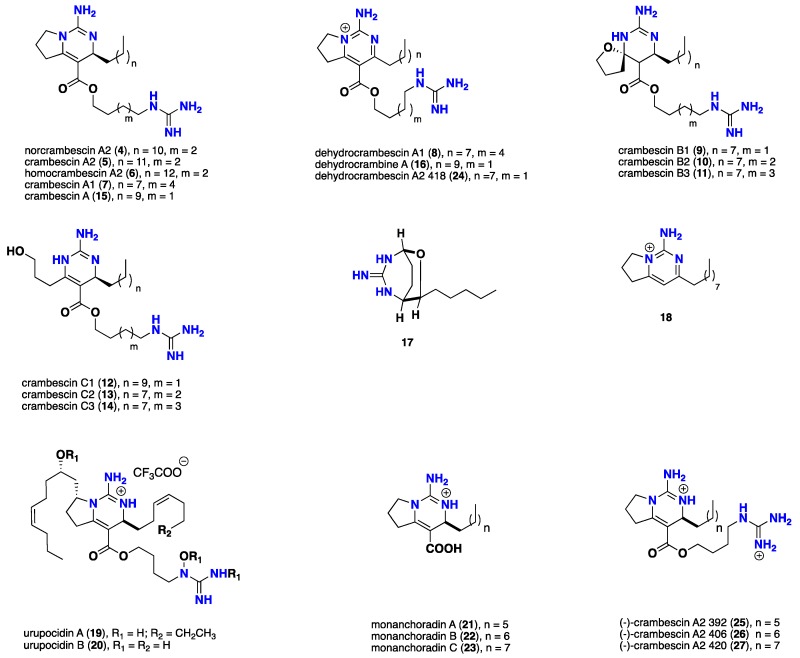
Isolated bicyclic guanidine alkaloids **4**–**27**.

Eleven bicyclic guanidine metabolites including five bearing crambescin type A (**4**–**8**), three bearing crambescin type B (**9**–**11**) and further three possessing crambescin type C (**12**–**14**) were recorded from the Mediterranean sponge *Crambe crambe*. Their structures were established using NMR and careful HRMS/MS data analyses for the complete assignment of the alkyl chain lengths. These compounds demonstrated cytotoxic activity against neuronal cell lines in micromolar range [34,40,41]. Additional homologue crambescin A (**15**), the only known bicyclic compound reported from the Caribbean sponge *Batzella* sp. Compound **15** displayed potent cytotoxicity against proliferating Vero cells and HIV gp120-human CD4 binding inhibition activity with IC_50_ > 100 μM [14]. Further bicyclic compounds including dehydrocrambine A (**16**) recorded from *Monanchora* sp. that inhibits HIV-1 fusion [42]. Monanchorin (**17**), a guanidine alkaloid with unusual bicyclic skeleton from *Monanchora ungiculata* showed very weak cytotoxic activity with IC_50_ = 11.3 μM against IC2 murine mast cell lines [35]. The simple pyrimidine monalidine A (**18**), an anti-parasitic bicyclic guanidine alkaloid, was recently recorded from *Monanchora arbuscula* [43]. Urupocidins A (**19**) and B (**20**), bisguanidine alkaloids possessing unusual *N*-alkyl-*N*-hydroxyguanidine motif, were isolated from *Monanchora pulchra*. Urupocidin A (**19**) increases nitric oxide production in murine macrophages via inducing iNOS expression [44]. Recently, seven cytotoxic guanidine alkaloids were described from a French Polynesian *Monanchora* n. sp. including three bicyclic architectures possessing a free carboxylic acid group monanchoradins A–C (**21**–**23**) and four bicyclic compounds bearing crambescin A2 type skeleton with a short butyl-guanidine side chain including dehydrocrambescin A2 418 (**24**), (−)-crambescin A2 392 (**25**), (−)-crambescin A2 406 (**26**) and (−)-crambescin A2 420 (**27**) along with monalidine A (**18**). Most of these compounds showed antiproliferative and cytotoxic activities against several cancer cell lines including KB, HCT-116, HL-60, MRC-5 and B16-F10, with IC_50_ values in the micromolar range. The bicyclic analogue monanchoradin A (**21**) that bearing a carboxylic acid functionality was found to be less potent, however, it is still in the nanomolar range. On the other hand, the bicyclic compounds **24**–**27** bearing the butyl-guanidine terminus were found more potent, in particular (−)-crambescin A2 420 (**27**) that was found to be the most active with IC_50_ = 0.03 μM against KB cancer cell lines [39]. Moreover, the simple compound **18** showed potent antiproliferative and cytotoxic activities against KB, HCT-116, MDA-435, HL-60 and MRC-5 with an IC_50_ values 0.2/0.4, 0.84/0.74, 0.32/0.86, 1.3/1.3, 0.55/0.60 μM respectively. It is worth noting that the bicyclic (−)-crambescin compounds **25**–**27** are enantiomers for the antipodal bicyclic (+)-crambescins, recently isolated from the marine sponge *Pseudaxinella reticulata* (now known as *Dragmacidon reticulatum*, family Axinellidae) and their recording draws important insights about chirality and its dependence on the species of sponge [45] (Figure 2).

### 2.3. Tricyclic Guanidine Alkaloids Bearing Ptilocaulin

**Figure 3:**
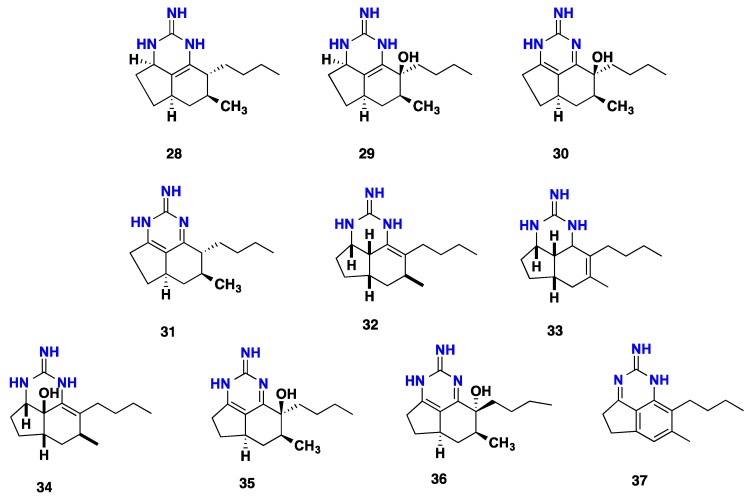
Isolated tricyclic guanidine alkaloids **28**–**37**.

Four tricyclic compounds including 8a,8b-dehydroptilocaulin (**28**), 8a,8b-dehydro-8-hydroxyptilocaulin (**29**), 1,8a;8b,3a-didehydro-8-hydroxyptilocaulin (**30**) and mirabilin B (**31**) were recorded from the Bahamas marine sponge, *Batzella* sp. [46]. (+)-Ptilocaulin (**32**), an antimicrobial and cytotoxic tricyclic guanidine alkaloid, in addition to isoptilocaulin (**33**) and (+)-8-hydroxyptilocaulin (**34**), were obtained from *Monanchora arbuscula* [38,47]. Moreover, (+)-ptilocaulin (**32**), exhibited antimicrobial activity against an oxacillin-resistant strain of *Staphylococcus aureus* with IC_50_ = 1.3 μM [48]. Further three tricyclic guanidine alkaloids, including 1, 8a; 8b, 3a-didehydro-8β-hydroxyptilocaulin (**35**), 1, 8a; 8b, 3a-didehydro-8α hydroxyptilocaulin (**36**) and mirabilin B (**31**), were described from *Monanchora unguifera* [49]. The mixture of **35** and **36** was active against the malaria parasite *Plasmodium falciparum* with an IC_50_ = 3.8 μM. Furthermore, mirabilin B (**31**) exhibited antifungal activity against *Cryptococcus neoformans* with an IC_50_ = 7.0 μM and antiprotozoal activity against *Leishmania donovani* with an IC_50_ = 17 μM [49]. The tricyclic guanidines **31**–**36** were identified from a Brazilian specimen of *Monanchora arbuscula* and were tested for their cytotoxicity against four cancer cell lines including HL-60, MDA-MB-435, HCT-8 and SF-295. The two compounds (+)-ptilocaulin (**32**) and (+)-8-hydroxyptilocaulin (**34**) displayed cytotoxicity with IC_50_ values ranging from 5.8–40.0 and 7.9–61.5 μM respectively. However, the other compounds **31**, **35** and **36** exhibited no activity. Additionally, compounds **32** and **34** were tested for their hemolytic activity against potential damage of mouse erythrocytes plasma membrane, where they displayed effective concentrations with EC_50_ values of 577.95 and 352.91 μM respectively [50]. Further anti-parasitic tricyclic guanidine alkaloid arbusculidine A (**37**) was reported recently from *Monanchora arbuscula* [43] (Figure 3).

### 2.4. Tricyclic Pyrroloquinoline Alkaloids

**Figure 4:**
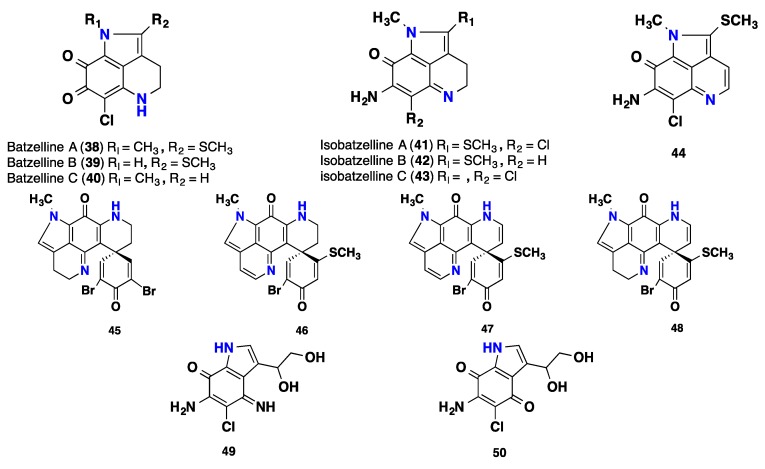
Isolated pyrroloquinoline alkaloids **38**–**50** from *Batzella* sp.

Seven highly functionalized pyrroloquinoline alkaloids including three compounds named batzellines A-C (**38**–**40**) and four compounds named isobatzellines A-D (**41**–**44**) were isolated from the deep-water Bahama’s sponge *Batzella* sp. The isobatzellines A-D (**41**–**44**) showed in vitro cytotoxicity against P388 leukemia cell with IC_50_ values 0.42, 2.6, 12.6 and 20 μM and moderate antifungal activity against *Candida albicans* with IC_50_ 3.1, 25, 50 and 25 μM respectively [7,51,52]. Further brominated compounds incorporating the pyrroloiminoquinone moiety, trivially named discorhabdins P, S, T and U (**45**–**48**) were obtained from a deep-water marine sponge of the genus *Batzella*. Discorhabdin P (**45**) inhibited CaN and CPP32 with IC_50_ values of 0.55 and 0.37 μM respectively. It also showed in vitro cytotoxicity against the cultured murine P-388 tumor cell line and human lung carcinoma A-549 cell line, with IC_50_ values of 0.025 and 0.41 μM, respectively [53]. Compounds **46**–**48** displayed in vitro cytotoxicity against cultured murine P-388 tumor cells, with IC_50_ values of 3.08, >5 and 0.17 μM, respectively. Further cytotoxicity was also observed for A-549 human lung adeno-carcinoma cells, with IC_50_ values of >5, >5 and 0.17 μM and for PANC-1 human pancreatic cells with IC_50_ values of 2.6, 0.7 and 0.069 μM, respectively [54]. A comprehensive review on their therapeutic applications has been reported [55]. Additionally, secobatzellines A-B (**49**–**50**), two simple pyrroloiminoquinone enzyme inhibitors were recorded from a deep-water marine sponge of the genus *Batzella*. Secobatzelline B (**50**) is an artifact compound that was obtained during the purification process. Secobatzelline A (**49**) inhibited calcineurin (CaN) and CPP32 with IC_50_ values of 0.55 and 0.02 μM. Moreover, secobatzelline B (**50**) inhibited calcineurin (CaN) IC_50_ values of 2.21 μM. Furthermore, compounds **49** and **50** displayed cytotoxicity in vitro against the cultured murine P-388 tumor cell line, with IC_50_ values 0.06, 1.22 μM and against human lung carcinoma A-549 cell line, with IC_50_ values of 0.04, 2.86 μM [56]. A huge number of synthetic aminoiminoquinone and aminoquinones analogues were prepared and tested as capase inhibitors [57]. Furthermore, a comprehensive evaluation for the cytotoxic activity of compounds **38**–**39**, **41**–**44** and **49**–**50** were determined against four different pancreatic cell lines Panc-1, AsPC-1, BxPC-3 and MIA-PaCa2 as well as in the Vero cell line, an epithelial cell line from the kidney tissue of an African green monkey [58] (Figure 4).

### 2.5. Polycyclic Alkaloids Bearing Batzelladine 

**Figure 5:**
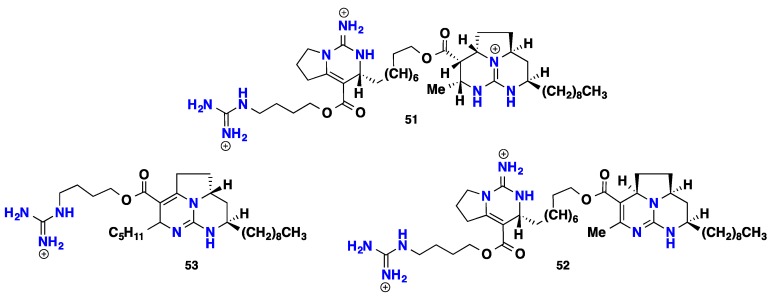

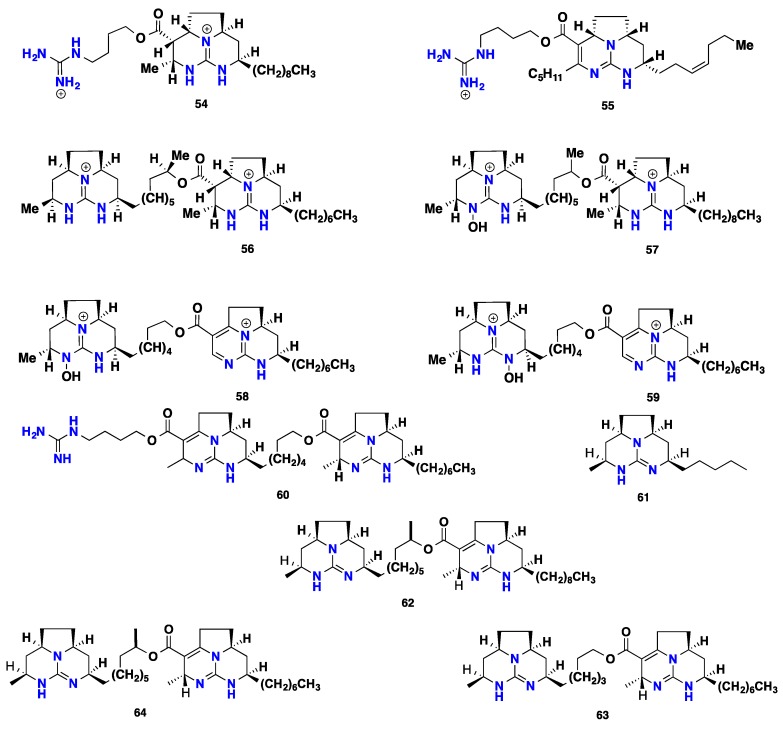
Isolated batzelladine alkaloids **51**–**64.**

**Figure 6:**
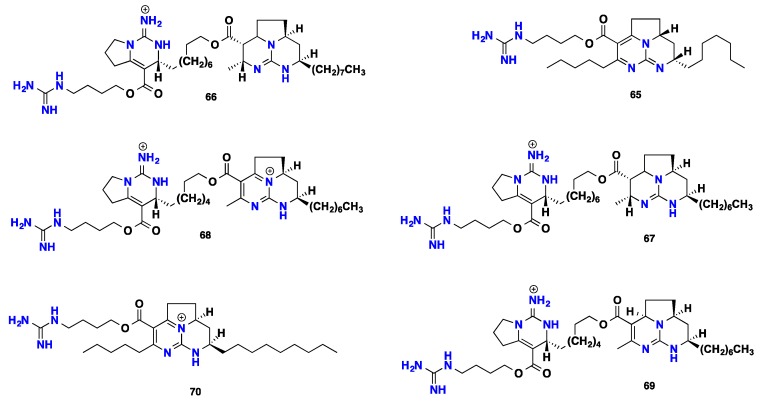

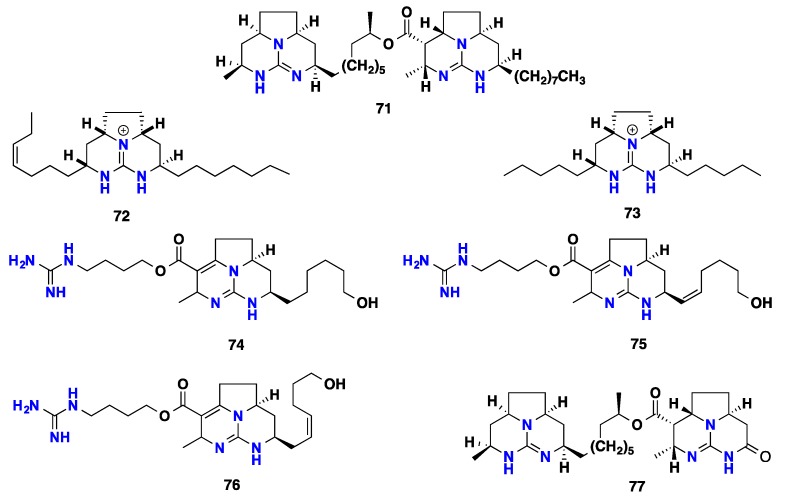
Isolated batzelladine alkaloids **65**–**77**.

Batzelladines represent a distinct class of particular guanidine-derived alkaloids that usually contain two main guanidinic moieties. Chemically, they are esters compounds that bear a principle tricyclic ring system named clathriadic acid that acting as an acidic portion bonded to another clathriadic acid molecule or crambescin A bicyclic system as an alcoholic part. Such a unique class of marine alkaloids is assumed to be synthesized biomimetically from different modes of cyclization between a polyketide-derived chain and a putative guanidine precursor affording these structurally complex metabolites [59]. These natural compounds are known for their potent bioactivities [13,14]. A considerable number of bioactive batzelladines were recorded from *Batzella* sponges. Batzelladines A-E (**51**–**55**), five potential inhibitors of HIV gpl20-human CD4 binding were recorded from the Caribbean sponge *Batzella* sp. [13,14]. Batzelladines F-I (**56**–**59**), four inducers of p56lck-CD4 dissociation, were isolated from *Batzella* sp. collected from Jamaica [60]. Batzelladine J (**60**) was isolated from the Caribbean *Monanchora unguifera* [61]. A further six guanidines—including batzelladines K-N (**61**–**64**), batzelladine C (**53**) and dehydrobatzelladine C (**65**)—were discovered from Jamaican *Monanchora unguifera* with activities against several cancer cell lines, protozoa, HIV-1 and AIDS [14,62,63]. Batzelladine C (**53**) displayed anti-HIV-1 activity at an EC_50_ of 7.7 μM [63]. Four batzelladines **66**–**69** containing crambescin A bicyclic system in addition to dihomodehydrobatzelladine (**70**) were reported from the Caribbean *Monanchora arbuscula*. These compounds displayed mild antitumor activity with GI_50_ (3–7 μM) against three cancer cell lines, lung carcinoma A549, colon carcinoma HT-29 and breast MDA-MB-231, in addition to antimalarial activity against protozoa [64]. Norbatzelladine L (**71**) was isolated from unidentified species, *Monanchora* sp. that displayed MNTC (maximum non-toxic concentration) at 2.5 μg mL^−1^ against HSV-1, with 97% of inhibition in the viral adsorption phase. Furthermore, it displayed cytotoxicity against several human cancer cell lines including leukemia, colorectal, breast, melanoma and glioblastoma [65,66]. Two anti-infective tricyclic members with unique stereochemical features—named merobatzelladines A–B (**72**–**73**)—were isolated from *Monanchora* sp. Merobatzelladines A-B exhibited moderate antimicrobial activity against *Vibrio anguillarum* with inhibitory zones of 9–10 mm on application of 50 μg of a sample to a paper disk of 6 mm diameter. Moreover, **72**–**73** also inhibited *Tripanosoma bruceibrucei* (GUT at 3.1) with IC_50_ = 0.24 μg mL^−1^ each. Furthermore, they display moderate inhibitory activity against the K1 strain of *Plasmodium falciparum* with an IC_50_ = 0.48 μM and 0.97 μM, respectively [67]. Four anti-parasitic batzelladines (**74**–**77**) against *Trypanosoma cruzi* and *Leishmania infantum* were recently recorded from *Monanchora arbuscula* [43,68]. Numerous synthetic batzelladines and their derivatives showed potent activities against HIV-1 and AIDS opportunistic infectious pathogens, inhibition of HIV-1 envelope-mediated fusion [69], inhibitors of HIV-1 Nef interactions with p53, actin and p56^lck^ [70], antimalarial, antileishmanial, antimicrobial and antiviral (HIV-1) activities [71], inhibitors against HIV-1 reverse transcriptase (RT) [72] and antileishmanial [73] (Figure 5 and Figure 6). 

### 2.6. Pentacyclic Alkaloids Bearing Crambescidin

**Figure 7:**
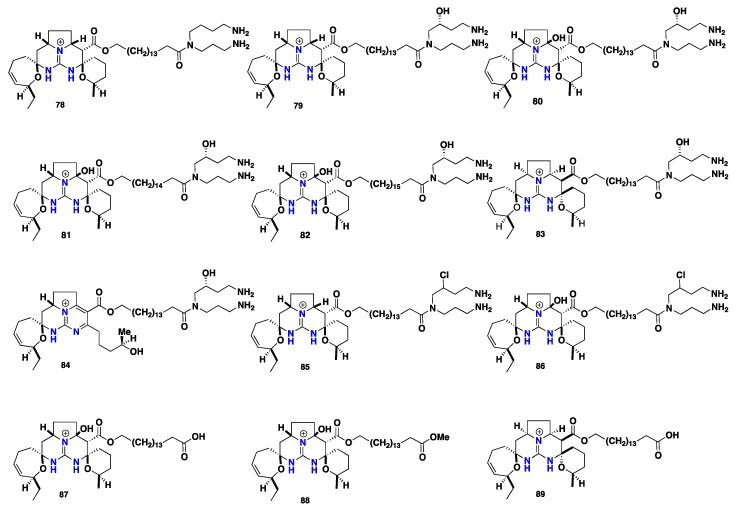
Isolated pentacyclic crambescidin alkaloids **78**–**89**.

**Figure 8:**
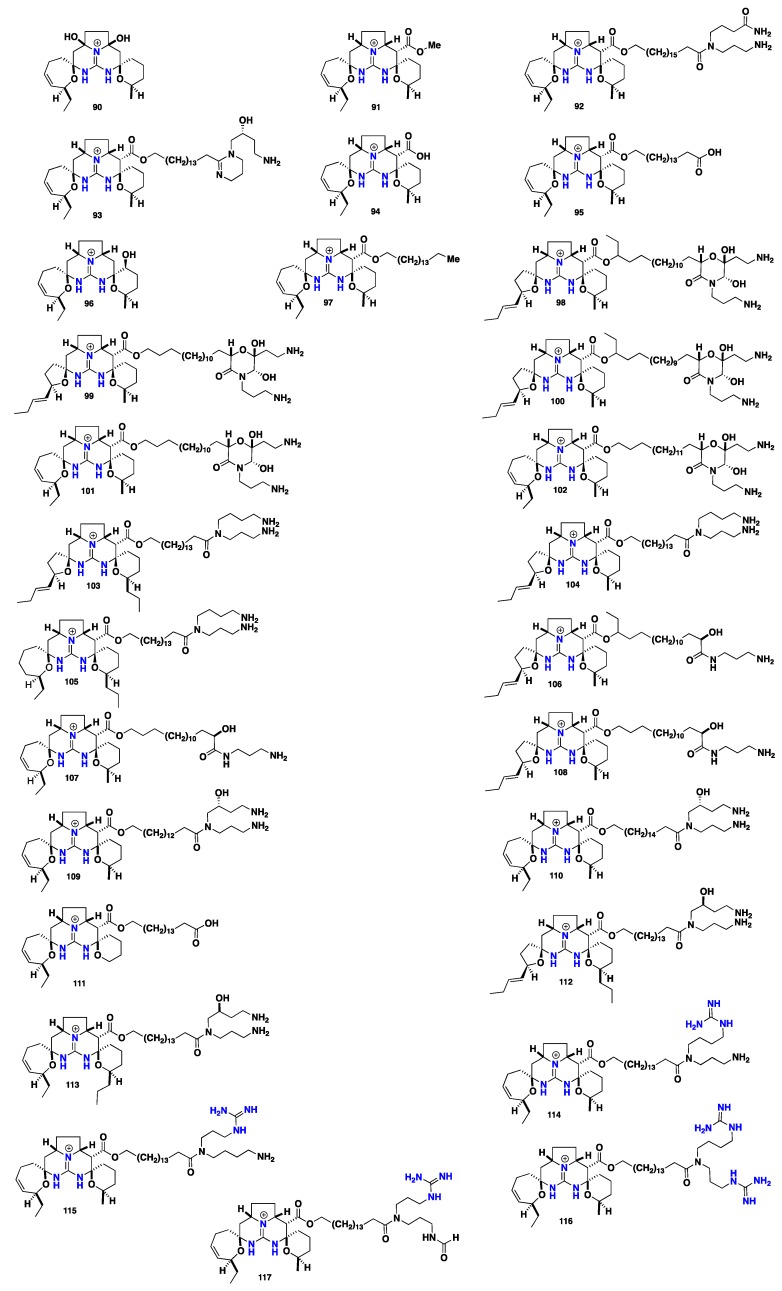
Isolated pentacyclic crambescidin alkaloids **90**–**117**.

Crambescidines are pentacyclic guanidine-derived alkaloids that represent recognizable complex marine metabolites. Chemically, they bear a common core of (5,6,8b)-triazaperhydroacenaphthalene in their molecules (trivially named as *vessel*) that coupled with a linear ω-hydroxy fatty acid (spermidine or hydroxyspermidine). These compounds vary from one to another in the length of the internal polymethylene chain and the oxidation degree of the two-spiro rings within the pentacyclic core. This group of compounds covers the major secondary metabolites recorded from these three genera. Since the discovery of the parent antiviral and cytotoxic marine metabolite ptilomycalin A (**78**) by Kashman and co-workers [74] from *Ptilocaulis spiculifer* (family Axinellidae) and *Hemimycale* sp. (family Hymedesmiidae) collected from the Red Sea coast in 1989, renewable efforts led to the discovery of further crambescidin analogues. Crambescidin 800 (**79**), crambescidin 816 (**80**), crambescidin 830 (**81**) and crambescidin 844 (**82**) were recorded from the Mediterranean marine sponge *Crambe crambe* [75]. These compounds demonstrated antiviral and cytotoxic activity against *Herpes simplex* virus, type1 (HSV-l) and cytotoxic activity against L1210 murine leukemia cells. Compounds **79**, **80** and **82** showed complete inhibition for HSV-l and 98% of L1210 cell growth at concentration of IC_50_ = 0.1 μM. Furthermore, crambescidin 816 (**80**) displayed potent Ca^2+^ antagonist activity and inhibited the acetylcholine-induced contraction of guinea pig ileum within very low concentrations [17], however, recent novel evidence showed that compound **80** partially blocked CaV and NaV channels in neurons, proposes that this compound might be included in decreasing the neurotransmitter release and synaptic transmission within the central nervous system [76]. Further, recent study proved that crambescidin 816 (**80**) could be stored into specialized sponge cells where it can be dispersed into the water affording a chemical umbrella surrounding the *Crambe crambe* sponge [77]. Recently, Botana and co-workers [78] reported important insights about the mechanism of the neurons cytotoxic activity of crambescidin 816 (**80**) in primary cultures of cortical neurons. These results showed that compound **80** is responsible for the decreasing of neuronal viability and hence provided a dose-dependent increase in cytosolic Ca^2+^ level that was also linked to the presence of Ca^2+^ in the extracellular media. Crambescidins **78**, **79** and **80** were recorded also from *Batzella* sp. [14]. 13,14,15-isocrambescidin 800 (**83**) with *trans*-ring junction within the pentacyclic core and crambidine (**84**) were discovered from *Crambe crambe* [17,79]. Surprisingly, compound **83** was found to be a less potent cytotoxic against L1210 cells compared to other crambescidines and there was no observed antiviral activity against HSV-1. This observation could be attributed to the enclosed ionic pocket feature found in **78** and related crambescidins and lacking in **83** [80]. Additional crambescidin analogues with a chlorinated spermidine motif including crambescidin 818 (**85**), crambescidin 834 (**86**), crambescidin 673 (**87**), crambescidin 687 (**88**) and 13,14,15-isocrambescidin 657 (**89**) without a spermidine unit were recorded from the FABMS guided isolation of *Crambe crambe* extracts. The ADMET predictor revealed that ptilomycalin and crambescidin 800 (**78**–**79**) possess three features of the Lipinski guidelines. Additionally, **78** showed low flexibility and a low tendency to permeate into cell membranes. However, compound **79** displayed low permeability, low flexibility and less tendency to permeate the cell membranes [81] Compounds **87**, **88** and **89** exhibited in vitro cytotoxicity against L1210 murine leukemia five times compared to compound **80**. Furthermore, they displayed antimicrobial activity against *Rhodotorula glutinis* [82,83]. Crambescidin 800 (**79**), crambescidin 359 (**90**) and crambescidin 431 (**91**) have been isolated from *Monanchora unguiculata* [62]. Crambescidin 826 (**92**) and fromiamycalin (**93**) were recorded from *Monanchora* sp. They inhibited HIV-1 envelope-mediated fusion in vitro with an IC_50_’s = 1–3 μM [14,42]. Indeed **78**, **79** and **93** displayed high cytotoxic activity against CEM 4 infected by HIV-1 with CC-50 of 0.11 μg mL^−1^, without cytoprotective effects, at a dose of <0.1 μM [84]. The antifungal **78** inhibits melanogenesis of *Cryptococcus neoformans* in vitro through the inhibition of the biosynthesis of laccase in the melanin biosynthetic pathway with an IC_50_ value of 7.3 μM [85]. Additionally, **79** induced a morphological change with neurite outgrowth in neuro 2A cells at concentration of 0.03–0.1 μM and recorded to induce the differentiation of K562 chronic myelogenous leukemia (CML) cells into erythroblasts accompanied by cell cycle arrest at the S-phase as well [86]. Further pentacyclic members were described, including crambescidin acid (**94**) from *Monanchora ungiculata* [35] and crambescidic acid (**95**) from *Monanchora unguifera* [61]. Crambescidin 359 (**90**) and 16-β-hydroxycrambescidin 359 (**96**) were obtained from *Monanchora unguifera* [63]. Ptilomycalin D (**97**) showed cytotoxicity against cancer cell line P-388 with IC_50_ = 0.1 μM in addition to **78** and **95** were reported from *Monanchora dianchora* [36]. Monanchocidins A-E (**98**–**102**) are five unusual pentacyclic guanidine alkaloids with a morpholine modified spermidine motif from *Monanchora pulchra*. These compounds exhibited potent cytotoxic activities against HL-60 human leukemia cells with IC_50_ values of 540, 200, 110, 830 and 650 μM respectively [37]. Monanchocidin A (**97**) showed anti-migratory activity against several human cancer cell lines where it is able to prevent local expansion and metastatic spread of cancer cells [87]. Moreover, it could be a promising new compound for overcoming resistance to standard therapies in genitourinary malignancies by the induction of autophagy and lysosomal membrane permeabilization [88]. Monanchomycalins A-B (**103**–**104**), two pentacyclic with a modified spiro five-membered ring, showed potent cytotoxicity against HL-60 human leukemia cells with the IC_50_ values 120 and 140 nM, respectively, were isolated from *Monanchora pulchra* [89]. Recently, compound **104** was recorded to inhibit of the TRPV1, TRPV2 and TRPV3 channels with EC_50_ values 6.02, 2.84 and 3.25 μM, respectively, however it displayed no activity against the TRPA1 receptor [90]. Moreover, monanchomycalin C (**105**) exhibited cytotoxicity against human breast cancer cell lines MAD-MB-231 with an IC_50_ of 8.2 μM, isolated from *Monanchora pulchra* [91]. Normonanchocidins A-B and D (**106**–**108**) were isolated from *Monanchora pulchra*. Compound **106** and a mixture of **107** and **108** (1:1) displayed cytotoxic activities against human leukemia THP-1 cells with IC_50_ values of 2.1 μM and 3.7 μM and against cervix epithelial carcinoma HeLa cells with IC_50_ of 3.8 μM and 6.8 μM, respectively [92]. Recently, further three cytotoxic pentacyclic guanidine compounds including crambescidin 786 (**109**), crambescidin 814 (**110**) and 20-norcrambescidic acid (**111**) along with pentacyclic analogues **79**, **90**, **92** and **95** were isolated from a French Polynesian sponge *Monanchora* n. sp. The isolated compounds showed potent antiproliferative and cytotoxic activities against KB, HCT-116, HL-60, MRC-5 and B16-F10 cancer cells. Compounds **109**, **110** and **111** exhibited cytotoxicity against KB cell lines with an IC_50_ values 0.3 μM, 5 nM and 0.5 μM, respectively. The two crambescidin **95** and **111** where the (*anchor*) motif is terminated with the carboxylic acid functionality displayed potent cytotoxic activity against KB cell lines with IC_50_ = 0.55 μM, however, they still less active compared with analogues possessing spermidine terminus. Furthermore, crambescidin 800 (**79**) exhibited the highest cytotoxic activity, while shorter pentacyclic homologue **109** along with the longer one **110** were found less active. These observations might highlight the impact of the polymethylene chain length within the (*anchor*) motif as a spacer for two site interactions. Crambescidin 359 (**90**), possessing only a pentacyclic core, showed no activity against KB cell lines and this correlates with the importance of the spermidine part for cytotoxicity. Regarding the B16-F10 murine melanoma cells, crambescidins **79**, **92** and **110** exhibited moderate activity with IC_50_ values of 0.2, 0.8 and 0.2 μM respectively. The discovery of 20-norcrambescidic acid (**111**) with this new pentacyclic motif carries some biogenesis impacts and raises some important insights about the variation in the oxidation degree and the mode of cyclization within the pentacyclic core [39]. A further two new hybrid pentacyclic guanidines monanchoxymycalin A-B (**112**–**113**) were obtained from the Far-Eastern marine sponge *Monanchora pulchra.* They displayed cytotoxic activities against cervical epithelioid carcinoma HeLa cells and breast adenocarcinoma MDA-MB231 cells [93]. Additionally, ptilomycalins E-H (**114**–**117**)—with guanidinic modified spermidine—were recorded from the Madagascar marine sponge *Monanchora unguiculata*. They displayed promising antimalarial activity against *Plasmodium falciparum* with IC_50_ values 0.38, 0.30 and 0.27 μM respectively [94,95] (Figure 7 and Figure 8). 

### 2.7. Acyclic Guanidine Alkaloids

**Figure 9:**
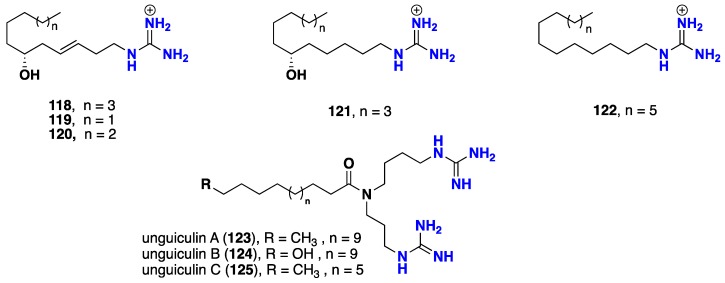
Isolated acyclic guanidine alkaloids **118**–**125**.

Small number of open chain guanidine-derived alkaloids was recorded. Pulchranin A (**118**), was described as the first marine non-peptide inhibitor of TRPV-1 channels with an EC_50_ value 41.2 μM, in addition two other acyclic members pulchranins B–C (**119**–**120**) reported from the Far-Eastern marine sponge *Monanchora pulchra*. Compounds **119** and **120** exhibited moderate inhibition against TRPV1 with EC_50_ value 95 and 183 µM respectively and were even less potent against TRPV3 and TRPA1 receptors [96,97]. Moreover, two synthetic derivatives—dihydropulchranin A (**121**) and hexadecylguanidine (**122**)—were prepared and studied for their TRPV channel-regulating activities. Compound **121** showed activity as an inhibitor of rTRPV1 and hTRPV3 receptors with EC_50_ values of 24.3 and 59.1 μM, respectively, while compound **122** was found not active against those receptors [98]. Additionally, recent studies revealed that pulchranin A (**118**) exhibited cytotoxic properties and prevented EGF-induced neoplastic transformation in vitro [99]. Further, acyclic analogue unguiculin A (**123**) with a modified bis-guanidine spermidine motif was isolated from the Madagascar marine sponge *Monanchora unguiculata*. It displayed antimalarial activity against the parasite *Plasmodium falciparum* with IC_50_ value of 6.04 μM [94,95]. Recently, a further two acyclic bis-guanidine alkaloids—named unguiculins B-C (**124**–**125**), beside unguiculin A (**123**)—were discovered from the French Polynesian *Monanchora* n. sp. sponge. These compounds displayed potent cytotoxic activity against KB cell lines with IC_50_ values 0.19/0.22, 0.08/0.09 and 0.03/0.03 μM respectively. Such activity might be attributed to the two terminal guanidines ends. Moreover, unguiculin C (**125**), the shorter homologue was found the most active. This could be concluded of how the chain and its length can play an important role as a spacer between two sites of interaction. Moreover, unguiculin B (**124**) showed further cytotoxicity against HCT-116, HL-60 and MRC-5 cell lines with IC_50_ values 3.6/3.6, >10/>10 and 9.6/11.4 µM respectively [100,101] (Figure 9).

### 2.8. Terpenoid Compounds

**Figure 10:**
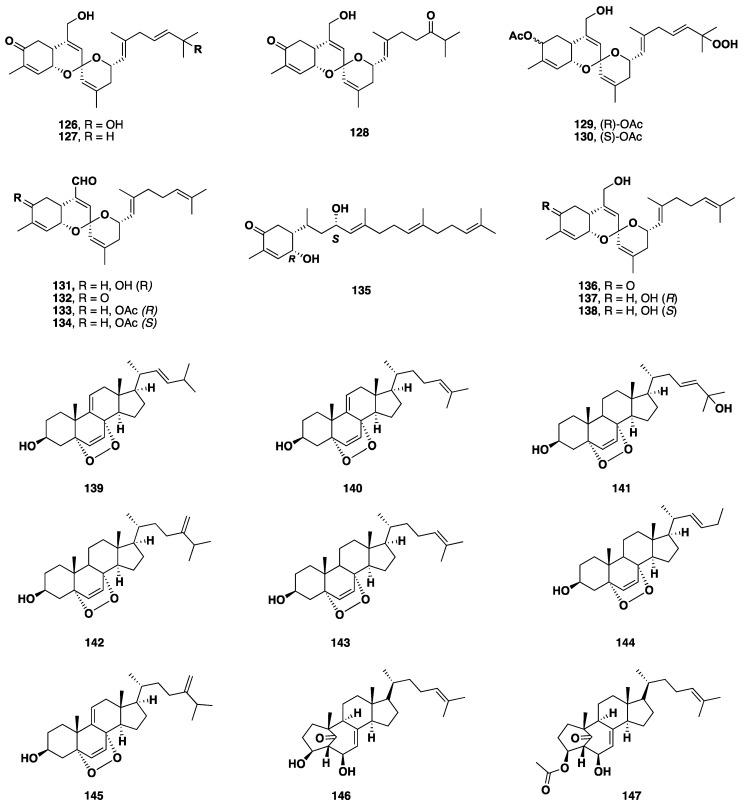
Isolated terpenoid and steroidal metabolites **126**–**147** isolated from *Monanchora* sp.

Marine sponges belong to *Monanchora* genus have also produced a small number of terpenoid metabolites and classical sterols [102]. Nine sesterterpenoids **126**–**134** were isolated from the Korean *Monanchora* sp. along with four phorbaketals **135**–**138**. These compounds were investigated for their cytotoxic activity against four human cancer cell lines—A498, ACHN, MIA-paca and PANC-1—where some of them showed potent cytotoxicity [103]. Seven cytotoxic 5α,8α-epidioxy sterols **139**–**145** were also described from *Monanchora* sp. These sterols showed moderate cytotoxicity against several human carcinoma cell lines including renal (A-498), pancreatic (PANC-1 and MIAPaCa-2) and colorectal (HCY-116) cancer cell lines [104]. Monanchosterols A-B (**146**–**147**) were identified from a South Korean *Monanchora* sp. and described as the first examples of naturally occurring steroids bearing a rearranged bicyclo [4.3.1] A/B ring system. Moreover, Monanchosterols A-B (**146**–**147**) exhibited significant inhibition of mRNA expression of Il-60 without notable cytotoxicity to the cells in a dose-dependent manner [105] (Figure 10). 

## 3. Biomimetic Landmarks of Polycyclic Guanidinium Motifs 

**Figure 11:**
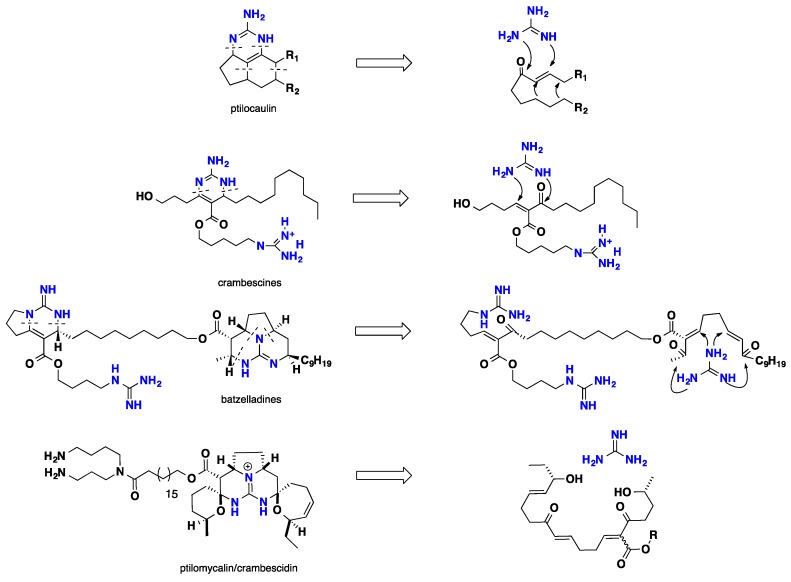
Structural analysis of different polycyclic guanidine alkaloids.

The bio-mechanistic studies along with the structural analyses for the different polycyclic guanidine alkaloids revealed two important insights; the first is chemical; where they are sharing the same biogenesis routs. A second is ecological; where marine sponges that produced such metabolites could be systematically classified under the same order. Generally, the different polycyclic guanidinic moieties could be biomimetically synthesized by way of the double aza Michael strategy, by the addition of free guanidine to α, β unsaturated polyketide chains (Figure 11) [59]. 

### 3.1. Bicyclic Compounds Possessing Crambescins Type A, B and C

**Figure 12:**
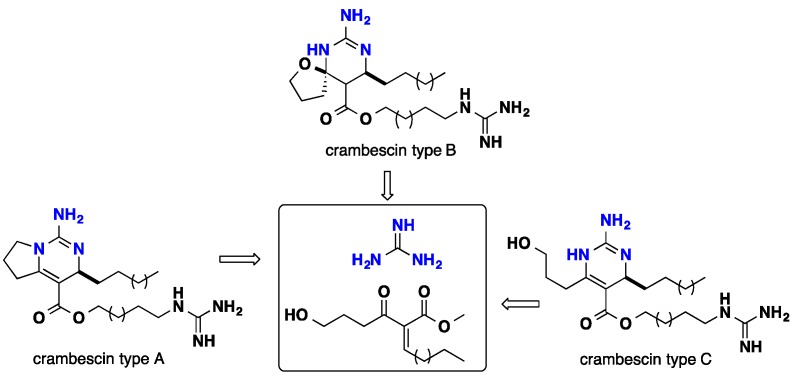
Proposed retrosynthetic analysis of the bicyclic alkaloids.

Snider and his team had several contributions towards the biomimetic synthesis of the polycyclic guanidinic motifs. The bicyclic crambescin alkaloids possess three different cyclic moieties—crambescin type A with tetrahydropyrrolo [1,2-c] pyrimidine nucleus, crambescin type B possesses an oxa-6,8-diazaspiro [4.5] motif, while crambescin type C displays a tetrahydropyrimidin fragment. Crambescins type B and C were isolated exclusively from the Mediterranean marine sponge *Crambe crambe* [34,40,41]. A postulated strategy showed that these three guanidinium cores could be constructed biomimetically through a conjugated Michael addition of guanidine to *enone* ester. This strategy seems pertinent since it gathers the formation of three different atom arrangements from one unified precursor (Figure 12) [106]. 

**Figure 13:**
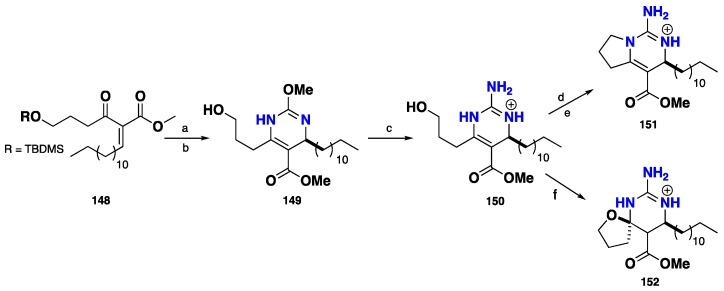
**a**: 2 equiv. *O*-methylisourea and 7 equiv. NaHCO_3_ in DMF for 12 h at 60 °C, 79%; **b**: hydrolysis, TBAF, THF, 12 h, rt, 90%; **c**: NH_4_OAc (1.5 equiv.), MeOH saturated with NH_3_ at 60 °C for 2 days, 61%; **d**: MsCl, Et_3_N in DCM for 30 min, 0 °C, 6 h, rt; **e**: Et_3_N in CHCl_3_, reflux, 12 h, 90%; **f**: Et_3_N in CHCl_3_, Δ, 12 h.

The less basic *O*-methylisourea was chosen as guanidine precursor instead of free guanidines. The condensation of *O*-methylisourea with previously prepared enone (**148**) followed by acid hydrolysis and desilylation afforded the corresponding dihydropyrimidine intermediate (**149**). In presence of methanolic ammonium acetate saturated with ammonia, **150** afforded the key compound **150**, corresponding to crambescin type C. Subsequently, **150** was transformed to compound **151**, corresponding to crambescin type A by mesylation, hydrogenolysis and cyclization. Compound **152** possesses crambescin type B was obtained by cyclization of **150** under basic condition (Figure 13) [106]. 

**Figure 14:**
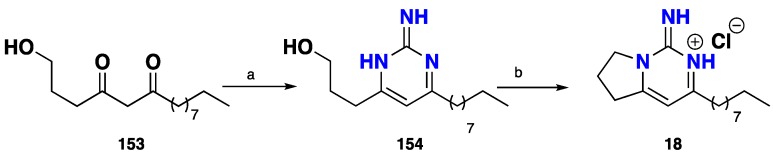
**a**: Guanidine hydrochloride, *t-*BuOK, CF_3_CH_2_OH, 30 min, then **154**, rt, 48 h, 25%; **b**: Ph_3_P, imidazole, I_2_, CH_2_Cl_2_, −18 °C, 6 h, 67%.

Based on the previous biomimetic approach, Berlinck and co-workers [43] accomplished the biomimetic synthesis of the cytotoxic and anti-parasitic monalidine A (**18**). 1,3-diketone **153** was introduced for condensation with guanidine free base to afford the corresponding pyrimidine **154** in 25% yield. Subsequently, the key intermediate **152** was cyclized using the Mitsunobu modified protocol to afford **18** as hydrochloride salt in a 67% yield (Figure 14).

### 3.2. Tricyclic Possessing Ptilocaulin/Batzelladine

**Figure 15:**
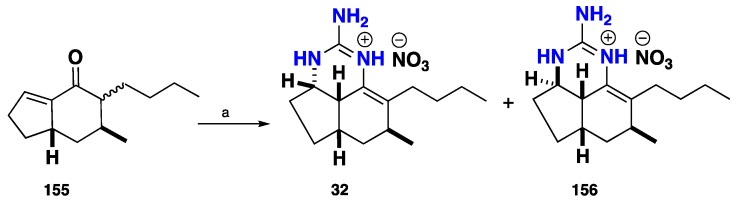
**a**: Guanidine, PH, reflux 25 h, then HNO_3_ (1% aq), 35%.

(±)-Ptilocaulin (**32**) was first synthesized biomimetically as a racemic mixture via Michael addition strategy by addition of free guanidine to *enone* **155** followed by intramolecular enamine formation. (−)-Ptilocaulin (**156**) was formed as a kinetic product where the guanidine was added to the less hindered top convex face of *enone* **155**, whereas (+)-ptilocaulin (**32**) was obtained as a thermodynamic adduct as the guanidine was added to the more hindered bottom side of *enone* **155**. This strategy highlights and proves a unique unified biosynthetic route for ptilocaulins and related tricyclic guanidinic analogues (Figure 15) [107,108,109]. 

**Figure 16:**
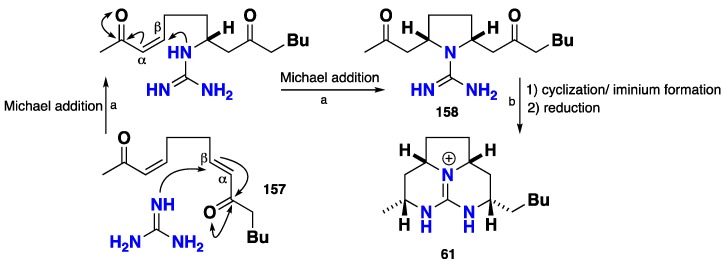
**a**: Guanidine, DMF, 0 °C, then 25 °C, 5 h **b**: 0 °C, MeOH-H_2_O (2:1), NaBH_4_ (6 equiv.), 25 °C, 16 h, 25%.

The tricyclic guanidinium framework of batzelladine K (**61**) was biomimetically synthesized through the addition of free guanidine to a *bis-enone* **157** affording the pyrrolidine-dione **158**, which was subsequently introduced to cyclization followed by iminium ion formation giving rise to the full fused tricyclic guanidinium core. A subsequent reduction afforded **61**. A unified synthetic strategy was applied to ptilocaulin (**32**), isoptilocaulin (**33**) and batzelladine K (**61**), which indicated that these classes of tricyclic guanidines are subjected to the same biomimetic gate (Figure 16) [71,110,111].

### 3.3. Pentacyclic Possessing Ptilomycalins, Crambescidins and Monanchomycalins

**Figure 17:**
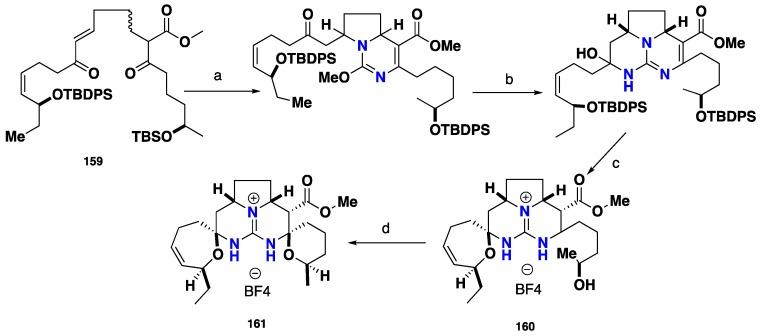
**a**: *O*-methylisourea, *i*-Pr_2_EtN, DMSO, 80 °C, 1.5 h (52%, 4:l, H_10_, H_13_ *trans*: H_10_, H_13_ *cis*); **b**: NH_3_, NH_4_OAc, *t*-BuOH, 60 °C, 40 h (72%, 1:1, H_10_, H_13_ *cis* β: H_10_, H_13_ *cis* α); **c**: 3:7 HF-CH_3_CN, −30 °C, 3d; **d**: Et_3_N, MeOH, 60 °C, 20 h (78%).

Numerous total syntheses of the pentacyclic guanidinium core of ptilomycalin A (**78**), crambescidin 800 (**79**) and crambescidin 359 (**95**) were biomimetically achieved [23,112,113]. A biomimetic synthesis of the methyl ester of the pentacyclic nucleus of **78** was conducted through a conjugated condensation of *O*-methylisourea as protected guanidine strategy with double Michael acceptor *bis-enone* **159** as α-β unsaturated polyketide framework. Subsequently, desilylation under acidic conditions provided the first seven-membered spiroaminal ring within the intermediate **160**. Later, the second six-membered spiroaminal ring was achieved under basic conditions followed by subsequently aminal formation affording the ptilomycalin A pentacyclic framework **161** (*vessel*) in one single biomimetic step (Figure 17).

**Figure 18:**
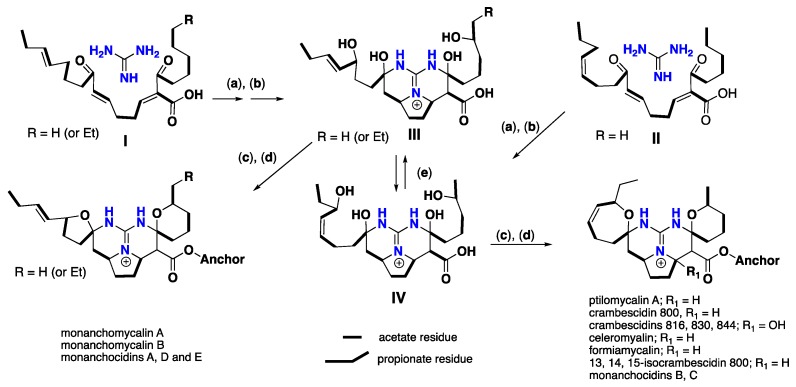
Proposed conversions (**a**–**e**) and hypothetical biogenesis of different pentacyclic guanidine alkaloids.

Recently, a detailed biomimetic gate was proposed illustrating the biogenesis of different pentacyclic guanidinium cores. The pentacyclic core of monanchomycalin A (**103**), suggests polyketide-like biogenesis, followed by spermidine-spermidine condensations. Two different precursors were employed, including either nine acetate units as in monanchomycalin B (**104**) and other known pentacyclic members, or ten acetate and one propionate units as in monanchomycalin A (**103**). To finish the pentacyclic guanidinium polyketide framework (*vessel*), a cyclization key-step developed by adding guanidine to *bis-*α, β unsaturated chain followed by imine-enamine tautomerization (transformation (**a**)). Further conversions including the allylic oxidation (transformation (**b**)) to afford putative intermediates (III and/or IV) followed by cyclization-elimination (**c**) and (**d**) to generate monanchomycalins A-B (103–104) and related pentacyclic analogues. Moreover, the interconversion of the presumptive intermediates III and IV (transformation (**e**)) through allylic rearrangement like reactions also might be possible (Figure 18) [89]. 

**Figure 19:**
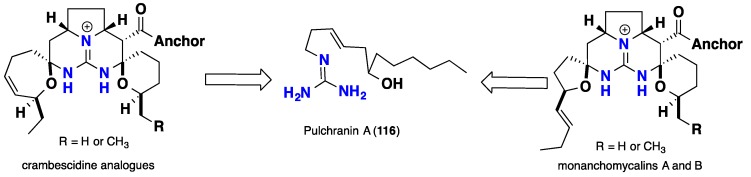
Pulchranin A (**118**), as a biosynthetic precursor for pentacyclic compounds (**103**–**104**).

Recently, Guzii and collaborators [96] proposed biogenetic correlations linking between the acyclic guanidine alkaloid pulchranin A (**118**) and the pentacyclic crambescidins and monanchomycalins A-B (**103**–**104**). This proposed biogenetic rout could unify the variation in the oxidation degree for the left-hand side spiroaminal rings (Figure 19).

## 4. Conclusions

In conclusion, we have presented complete and comprehensive up-to date literature survey exclusively dedicated to the chemistry, biology and insights on the most leading biomimetic syntheses of guanidine derived natural products isolated from marine sponges of three genera *Batzella*, *Crambe* and *Monanchora*. One hundred forty-seven marine natural products were recorded with distinct structural diversities that afforded wide scope of bioactivities. For their chemodiversity, along with their displayed biological potentialities, they still present promising and attractive marine species that are worth attracting the worldwide interest of natural products chemists and pharmacologists. 

## Abbreviations

The following abbreviations are used in this manuscript:

ADMET	absorption, distribution, metabolism and excretion–toxicity in pharmacokinetics
EC50	Half maximal effective concentration
GI50	Half maximal growth inhibition
gp120	glycoprotein 120
HIV-1	Human immunodeficiency virus 1
HSV-1	*Herpes simplex* virus 1
IC50	Half maximal inhibitory concentration
MIC	Minimum inhibitory concentration
